# Case of Hemorrhagic Diathesis

**Published:** 1881-02

**Authors:** 


### CASE OF HEMORRHAGIC DIATHESIS.

The following good example of this is given by Prof. George Buchanan, in the Glasgow *Medical Journal,* November, 1880: —

The patient, a young man, aged twenty-four, presents a strikingly anæmic appearance, and is so debilitated by protracted hemorrhage that he is quite confined to bed, becoming giddy if he sits up even for a short time. Four years ago he was admitted to the hospital on account of continued hemorrhage following the extraction of a tooth. The actual cautery was used on that occasion, and after a short residence he was dismissed apparently quite well. A few days later, however, a return of the hemorrhage occurred. Plugging with lint and perchloride of iron was resorted to, and it finally ceased. The account of the present occurrence is that, on the 29th of August last he had one of the upper left molars extracted by a dentist, to whom no mention was made of the previous troublesome hemorrhage; blood continuing to flow, a plug of lint and matico was applied, but with no very good effect. The plugs were renewed at intervals for a week, but failed thoroughly to control the hemorrhage. The patient then consulted Dr. Donald M'Phail, who, on September 5th cauterized the bleeding surface and plugged the cavity. This procedure appeared at the time successful, but on the same night oozing took place through the plug, and two days later the hemorrhage became more active, and continued intermittently for a week, when it stopped spontaneously. After two days it again commenced; a gutta percha plug was now applied, and with good effect, but the gum by this time had become so tender that the patient could not endure the necessary pressure, and he was therefore advised to enter the hospital, which he accordingly did on the 18th of September. On the 21st chloroform was administered, and Professor Buchanan removed the adjacent tooth and some dead bone in the neighborhood of the bleeding. The antrum of Highmore, which contained a considerable quantity of fetid pus, was then opened, a thick pyogenic membrane scraped out, the actual cautery applied, and the antrum stuffed with lint. In spite, however, of this very thorough treatment, the bleeding returned on the 26th, but was easily controlled by re-stuffing the antrum. On the 30th it again recurred. At this date subcutaneous injection of ergotine was begun, four grains being administered three times daily, as a rule, for about ten days, and afterwards less frequently. This appears to have had a good effect, as up to the date of writing (14th of October) there has been no return of the hemorrhage. During this period the plug was allowed to remain in the antrum, but ultimately it softened, and came away in shreds.

